# How many do we need? Meeting the challenges of studying the microbiome of a cryptic insect in an orchard

**DOI:** 10.3389/fmicb.2024.1490681

**Published:** 2025-01-06

**Authors:** Apolline Maurin, Audrey-Anne Durand, Claude Guertin, Philippe Constant

**Affiliations:** Centre Armand Frappier Sante Biotechnologie, Institut National de la Recherche Scientifique, Laval, QC, Canada

**Keywords:** *Conophthorus coniperda*, microbiome, sampling effort, cryptic insect, seed orchard’s pest

## Abstract

The minimal sampling effort required to report the microbiome composition of insect surveyed in natural environment is often based on empirical or logistical constraints. This question was addressed with the white pine cone beetle, *Conophthorus coniperda* (Schwarz), a devastating insect pest of seed orchards. It attacks and stop the growth of the cones within which it will spend its life, on the ground. To survive, the bark beetle probably interacts with microorganisms involved in alimentation, cold adaptation, and dormancy stage. Deciphering the drivers and benefits of these microorganisms in an orchard first requires methodological development addressing variability of the white pine cone beetle microbiome. The number of insect guts integrated in composite samples prior to DNA extraction and the number of surveyed trees are two features expected to induce variability in recovered microbiome profiles. These two levels of heterogeneity were examined in an orchard experimental area where 12 white pine trees were sampled and 15 cones from each tree were grouped together. For each tree, 2, 3 and 4 insects were selected, their intestinal tract dissected, and the microbiome sequenced. The number of insects caused no significant incidence on the coverage of bacterial and fungal communities’ composition and diversity (*p* > 0.8). There was more variability among the different trees. A sampling effort including up to 33 trees in an area of 1.1 ha is expected to capture 98% of the microbial diversity in the experimental area. Spatial variability has important implications for future investigations of cryptic insect microbiome.

## 1 Introduction

Insects live in close association with an array of microorganisms. Such interactions are essential for the protection, nutrition, and establishment of insects (Jang et al., 2024; Popa et al., 2012; Berasategui et al., 2016). Therefore, their microbiome is often studied to understand the crucial roles it plays in their survival to develop biological control techniques and/or assess the effects of the climate change or pollution on insect’s populations (Zhang et al., 2021; Li et al., 2021; Mariño et al., 2016; Jang et al., 2024). However, the methods of sampling and the types of samples considered in microbiome studies are often overlooked, leading to arbitrary choices in sample size selection (Prosser, 2010). For instance, three replicate samples representative of tested environments or experimental conditions are often selected as a trade-off between logistic efforts and the minimum number of observations for statistical analyses (Montgomery, 2006). Elaboration of experimental designs are mostly based on these limits, without accounting for the diversity and variability of microbial communities across the environment (Prosser, 2010; Knight et al., 2018). Conducting microbiome studies without considering the statistical power of the sample size is prone to failure of rejecting a false null hypothesis, while impairing generalization of research findings to broader contexts (Chow, 1988).

Here, *Conophthorus coniperda* (Schwarz) or white pine cone beetle (WPCB) is used as a case study for sample size and power calculations for the elaboration of an experimental design examining the relationship between microbiome composition and environmental conditions faced by the insect. This bark beetle is a significant pest in white pine cone orchards, where it targets and destroys cones, thereby impacting seed production and forest regeneration (Godwin and Odell, 1965; Guertin and Trudel, 2006). This cryptic insect develops and remains in cones on the ground surface over the winter season, before initiating a new life cycle in springtime (Henson, 1961). White pine appears to be the sole host of WPCB (de Groot et al., 1992), which make the seeds orchards ideal habitats for the establishment of insect populations. Seed orchards are thus suitable observatories to examine succession of WPCB microbiome across different scales encompassing orchard, tree or cone levels.

To date, there is no research on the microbiome of the WPCB. The small size of the insect making DNA extraction and amplification challenging, and the decoupling between the number of insects found in individual and cone size (Supplementary Figure 1) implies multiple considerations for the elaboration of robust experimental design. Hence, the aim of our study is to determine if the number of insect guts integrated in composite samples prior to DNA extraction and the number of surveyed trees are inducing variability in the recovery of microbiome profiles in a white pine seeds orchard. These results will guide future investigations examining the relationship between insect microbiome and environmental factors across different populations and sites. Ultimately, the acquisition of such knowledge could lead to the development of effective microbial ecological control strategies against this pest.

## 2 Materials and methods

### 2.1 Collection of white pinecone beetles

**Figure 1:**
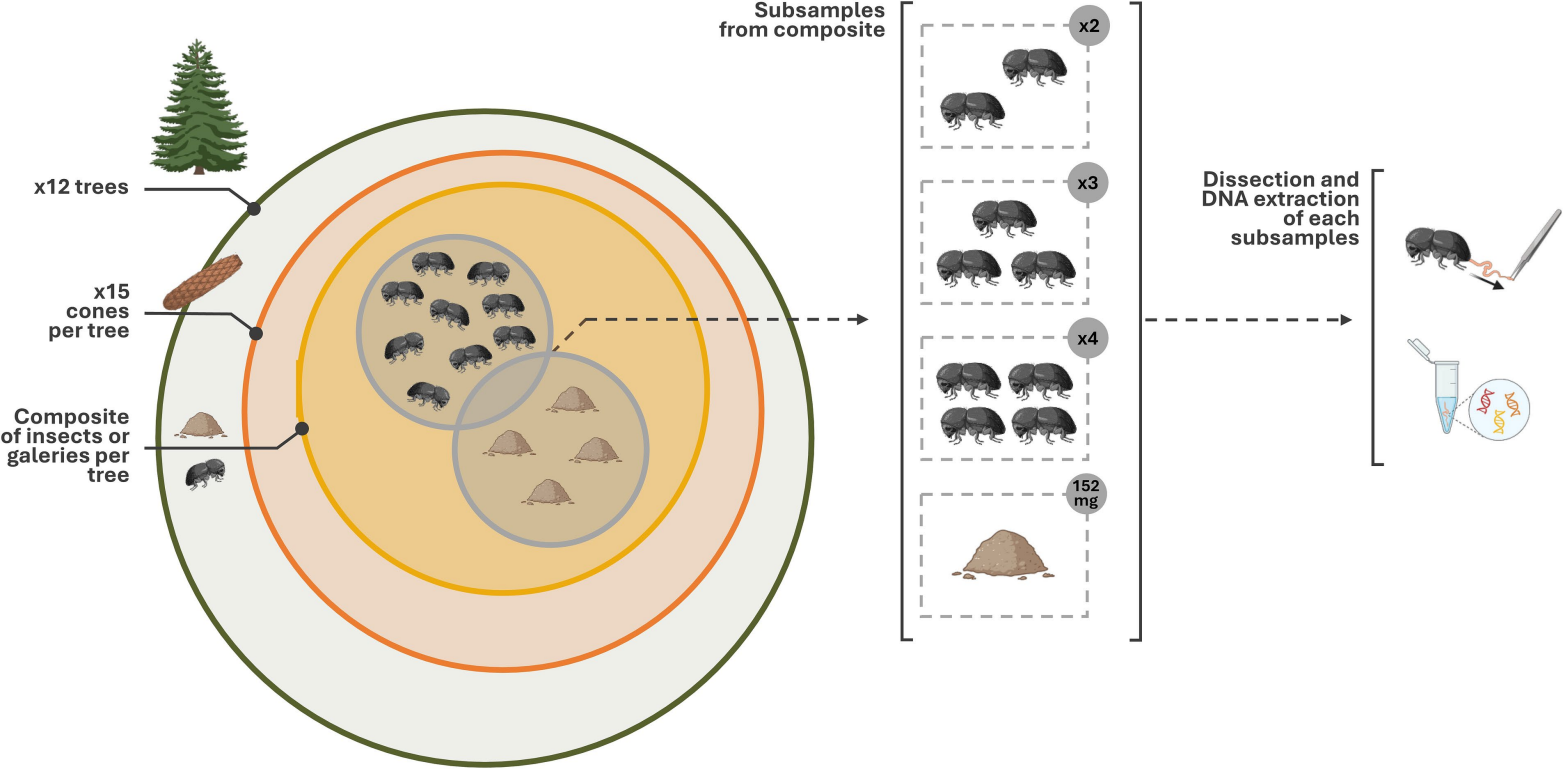
Graphical representation of the sampling method. Twelve trees have been selected. For each, 15 cones were sampled (~180 cones in total). Insects and galleries found in cones were distinctly pooled per tree (i.e., 12 pools). For galleries, after homogenisation, 152 mg were collected for each composite. For the insects, after sterilizing their surface, we subsampled two, three and four of their intestinal tracts. The number of sample sequenced varied because their concentration did not allow the sequencing of all sample (<3 ng/uL). The figure have been created using Biorender’s icons (https://BioRender.com/g02q920).

The insects were collected in the white pine seed orchard of Verchère, located in Saint-Amable (Québec, Canada, Lat. 45.677000, Long. −73.330000) with the permission of the Ministère des Ressources naturelles et des Forêts (MRNF). The orchard has a surface area of about 35,815 m^2^ and 3,758 trees spaced at 5 meters intervals. The MRNF has delimited six areas, each containing between 400 and 800 trees (Supplementary Table 1). Two areas available for field experimentations were utilized to evaluate the potential block effect (experimental area) and the incidence of individual trees, and number of trees on the diversity and the composition of microbiome associated with insect gut and galleries. Twelve trees have been randomly selected throughout area 2 and area 6 (1.1 ha; Supplementary Table 1). For each individual tree, 15 attacked cones were collected on the ground and kept in a paper bag in a cooler during transportation (Figure 1). They were then stored overnight at 4°C until processed. The cones were dissected to collect insects with sterile soft tweezers and the gallery biomass, comprising a mixture of frass and lignocellulosic residues, with a sterile micro-scoop. WPCB from the same cone were pooled in a single 1.5 mL sterile microcentrifuge tube and the galleries were placed in a distinct tube. The samples were stored at −80°C until processed.

### 2.2 Sample preparation and DNA extraction

Two different approaches were implemented to prepare gallery biomass and insect samples. The pooled galleries were homogenized, and 152 mg ± 4 mg was collected and placed in a 2 mL microcentrifuge tube containing 100 μL of extraction buffer (50 mM Tris-HCl, 5 mM EDTA-2Na, 3% SDS, pH 8.0). Gallery samples A5 and A6 were represented by 56 mg and 135 mg, respectively. For the insects, a representative sample of each tree was assembled by the random selection of 12 insects placed in a 2 mL microcentrifuge tube to pursue with dissection. First, the elytra and the wings of *C. coniperda* were removed using sterile tweezers and scissors under a stereoscopic microscope (Zeiss, Toronto, ON, Canada). Up to 7 wingless beetles were placed in a 1.5 mL microcentrifuge tube to ensure a proper sterilization. Surface sterilization comprised a first wash in 1 mL 70% ethanol (EtOH) with 1 min 30 s vortex mixing (Fisher Vortex Genie 2, Ottawa, ON, Canada) and two serial washes in 1 mL of sterile 1X phosphate-buffered saline (PBS) (137 mM NaCl, 2.7 mM KCl, 10 mM Na_2_HPO_4_, 2 mM KH_2_PO_4_, pH 7.4) with 1 min 30 s vortex mixing. The insects were then kept in 1 mL of sterile 1X PBS. The gut of each individual was recovered by cutting the last tergite and then gently pulling the tract until the intact midgut was exposed. Guts were assembled in different subsamples for each tree by pooling 2, 3 and 4 guts in a 2 mL microcentrifuge tube containing 100 μL of extraction buffer (Figure 1). Dissected guts were then kept at −20°C overnight until total DNA extraction. The number of tracts pooled was chosen according to the number of insects mainly found in cones (3 to 7; Supplementary Table 2) and the structural fragility of the WPCB. In this way, future analyses could concentrate on cone composite sample as much as the individual sample. Similarly, the number of replicates and cone used depended on the number of insects in cones (Supplementary Table 2).

The tracts were crushed with a pestle for microtube and vortexed for 1 min. Then, 700 μL extraction buffer and 5 μL proteinase K (800 U/mL) (New England Biolabs, Whitby, ON, Canada) were added to the tubes containing the guts or the galleries. After gently homogenizing, the tubes were placed 1 h at 55°C to activate the proteinase K, followed by heat inactivation at 95°C for 15 min. A volume of 2 μL of RNase (10 mg/mL) (Thermo Fisher Scientific Baltics UAB, Vilnius, Lithuania) was added and left to react at 37°C for 15 min. Total DNA was then extracted using the method described by Durand et al. (2015) with the following modifications. To have a better precipitation and visualization of the DNA pellet, 5 μL glycogen (400 μg/mL) (molecular biology grade; Thermo Fisher Scientific Baltics UAB, Vilnius, Lithuania) was added to the sample with the isopropyl alcohol (2-Propanol). In addition, the DNA precipitation was done overnight at −20°C. DNA samples were stored at −20°C until PCR amplicon sequencing library preparation.

### 2.3 PCR amplicon sequencing library preparation, sequencing and raw reads processing

PCR amplicons of the V3–V4 region of bacterial 16S rRNA gene were prepared with the primers 515f (5′-GTG CCA GCM GCC GCG GTAA-3′)—806r (5′-GGG ACT ACH VGG GTW TCT AAT-3′) (Caporaso et al., 2012) and PCR amplicons of the fungal ITS1 region were prepared with the primers ITS1 (5′-TCCGTAGGTGAACCTGCGG-3′) (White et al., 1990)—58A2r (5′-CTGCGTTCTTCATCGAT-3′) (Martin and Rygiewicz, 2005). Procedure to prepare the sequencing libraries is described in a previous work (Bourdin et al., 2021). Samples with a minimum concentration of 3 ng/μL were shipped to the Centre d’Expertise et de Services Génome Québec (Montréal, Québec, Canada) for Illumina Miseq PE-250 sequencing. The number of samples sequenced for each condition can be found in Supplementary Table 2. Raw sequence reads were deposited in the Sequence Read Archive of the National Center for Biotechnology Information under Bioproject PRJNA1146082. All the data processing and statistical analysis were performed on the R software (R Core Team, 2023). Raw sequencing reads were processed using the software Cutadapt v.3.5 (Martin, 2011) and the package Dada2 v.1.30.0 (Callahan et al., 2016). Taxonomic assignation of amplicon sequence variation (ASV) was done using the UNITE database v.9.0 (Abarenkov et al., 2022) for fungi and the Silva v.138.1 database (McLaren and Callahan, 2021) for bacteria and archaea. ASVs matching chloroplast and mitochondria sequences were removed. In addition, ASVs with frequencies <0.005% were discarded to reduce noise (Bokulich et al., 2013), with potential loss of rare, but ecologically relevant ASV. The bacterial ASV table comprised 2,788 ASVs before filtering and 477 ASVs after with 2,918,781 sequences. The fungi table is composed of 571 ASVs before filtering and 241 after with 1,166,768 sequences.

### 2.4 Statistical analysis

The bacterial and fungal composition of the galleries and intestinal tract of *C. coniperda* was evaluated by looking at the relative abundance and their core microbiome. Only ASVs meeting specifics criteria were considered in the analyses. For the relative abundance, a minimum of 3% was required and for the core microbiome, a prevalence of 0.001 in at least 75% samples. The analysis was performed using rarefied count. Common taxon between the insect’s tract and their galleries were represented with a Venn diagram using the package eulerr v.7.0.2 (Larsson, 2018).

Alpha diversity of the gut microbiome and galleries was examined with three Hill numbers (*q*) encompassing species richness (*q* = 0), the Shannon index (*q* = 1) and the Simpson index (*q* = 2). These numbers were interpolated with a reference sample size using the package iNext v.3.0 (Hsieh et al., 2016). Parametric (ANOVA followed by *post hoc* Tukey tests or Student’s *t*-test) or non-parametric (Kruskal–Wallis followed by *post hoc* Dunn tests) analyses were conducted with the package stats v.4.3.2 (R Core Team, 2023) to compare the alpha diversity between the number of tract and/or the galleries. Power analysis was conducted with the G*Power freeware (Faul et al., 2009) when *H*_0_ could not be rejected (Supplementary Table 2). The result was considered robust if the *β* error, i.e., the probability of wrongly accepting the null hypothesis, was under 20%.

Beta diversity was related to the number of tracts, trees, and experimental areas (blocks). Samples were aggregated in the reduced spaces defined by a principal coordinate analysis (PCoA) using Bray–Curtis and weighted UniFrac distances with the ordinate function of the physloseq v.1.46.0 package (McMurdie and Holmes, 2013). All distances were computed from Hellinger-transformed ASV abundance table (Legendre and Gallagher, 2001) using the function decostand and vegdist from the vegan v.2.6-4 package (Oksanen et al., 2022). The relationship between the number of tracts, trees, and blocks was tested with a permutational multivariate analysis of variance based on distance matrices (PERMANOVA) conducted with the adonis2 function in the vegan v.2.6-4 package (Oksanen et al., 2022). These analyses were performed with the tracts and galleries or the tracts only. Finally, an analysis of composition of microbiome with bias correction (ANCOM-BC) was performed to identify taxa differing in abundance in relation to a variability of interest using ancombc from the package ANCOMBC v.2.4.0 (Lin and Peddada, 2020).

Gamma diversity was computed to examine whether the sample effort was sufficient to properly represent gallery and insect microbiome from the experimental area of the seed orchard comprising two blocks. It was represented as a species accumulation curve: the number of observed ASVs compared to the sampling effort, i.e., the number of trees (Gotelli and Colwell, 2001). The accumulation curve was obtained using the specaccum function of the vegan v.2.6-4 package (Oksanen et al., 2022). The number of additional samples needed (i.e., trees) to detect 98% of the estimated asymptotic species richness was estimated with the nonparametric methods proposed by Chao et al. (2009) with the Excel spreadsheet provided by the authors.

## 3 Results

### 3.1 Bacterial and fungal composition of the galleries and intestinal tract of *Conophthorus coniperda*

**Figure 2:**
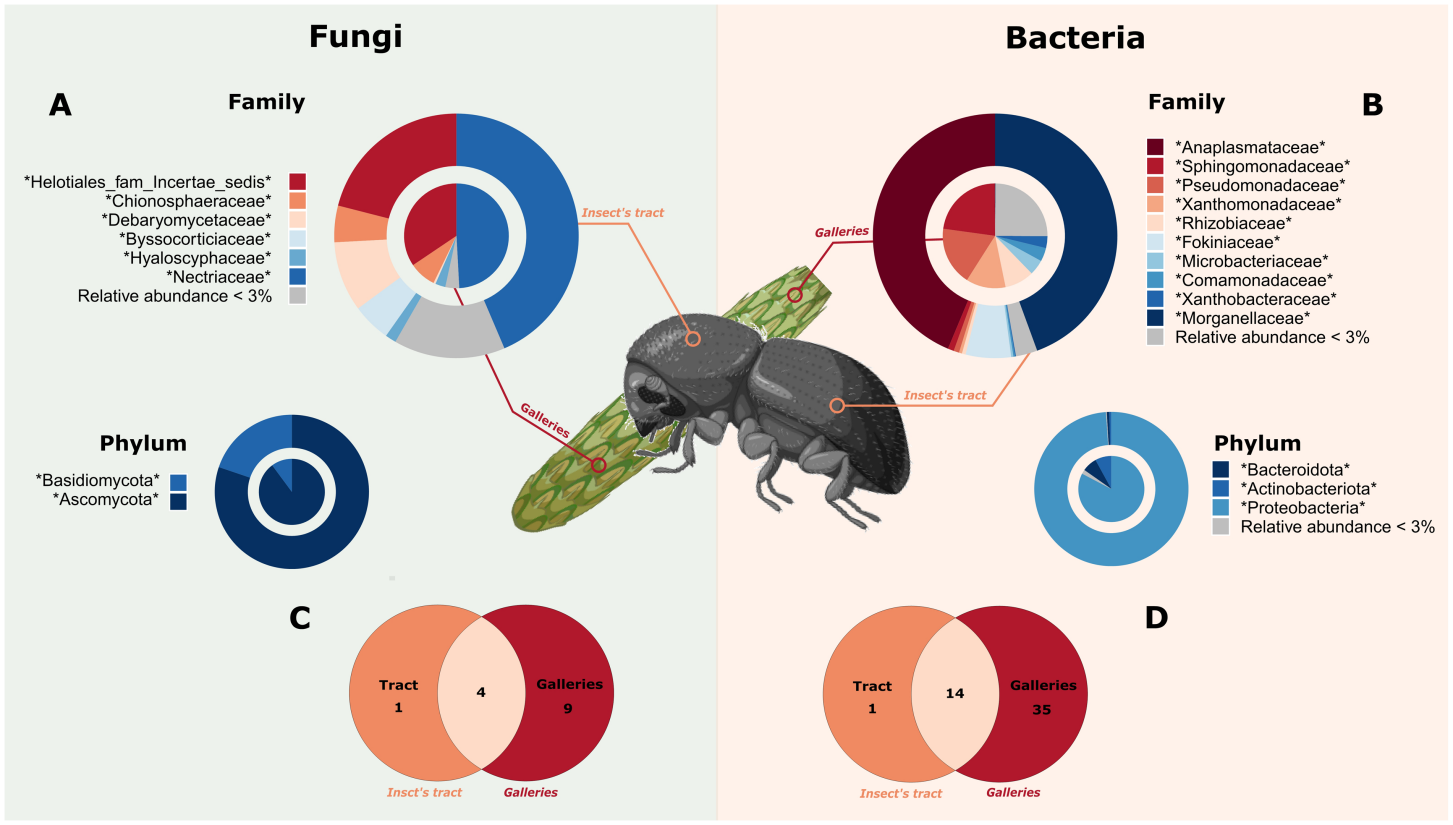
Microbiome composition of the white pine cone beetle and its galleries. The microbial composition of the insect is represented in the outer ring and in orange and/or the icon of an insect. The galleries’ communities are represented in the circle and in red and/or a cone. **(A)** Fungal composition at the family and phylum level. **(B)** Bacterial composition at the family and phylum level. Shared taxa among insect gut and galleries are represented with a Venn diagram for fungi **(C)** and bacteria **(D)**. The figure have been created using Biorender’s icons (https://BioRender.com/g02q920).

At the family level, both the intestinal tract of the beetle and the gallery were dominated by rare taxa. The mean relative abundance of family taxa inferior to 3% was represented by 95.95% bacterial and 90.54% fungal ASVs (Figure 2; Supplementary Table 2). However, in term of relative abundance, three taxa represented 94% of the bacteria found in the intestinal tracts of the insect (*Anaplasmatacea*, *Morganellaceae* and *Fokiniaceae*) and 53% of those detected in the galleries (*Sphingomonadaceae*, *Pseudomonadaceae* and *Xanthomonadaceae*). All these taxa belong to the phylum Proteobacteria. Fungi encompassed the phyla Basidiomycota and Ascomycota, the latter representing 85% relative abundance. Furthermore, at the family level, 14 bacterial ASVs and 4 fungal ASVs were shared between the insects and their galleries. Shared bacterial ASV represented 93% of ASV detected in insects and 32% of ASV detected in galleries, whereas shared fungal ASV represented 80 and 29% of ASV detected in insects and galleries, respectively. These shared taxa were mainly from Proteobacteria and Ascomycota. The ASVs present in at least 75% of the samples and the shared ASVs can be found in the Supplementary Table 2.

### 3.2 The number of insect’s guts does not influence the microbiome profile

#### 3.2.1 Alpha diversity

**Figure 3:**
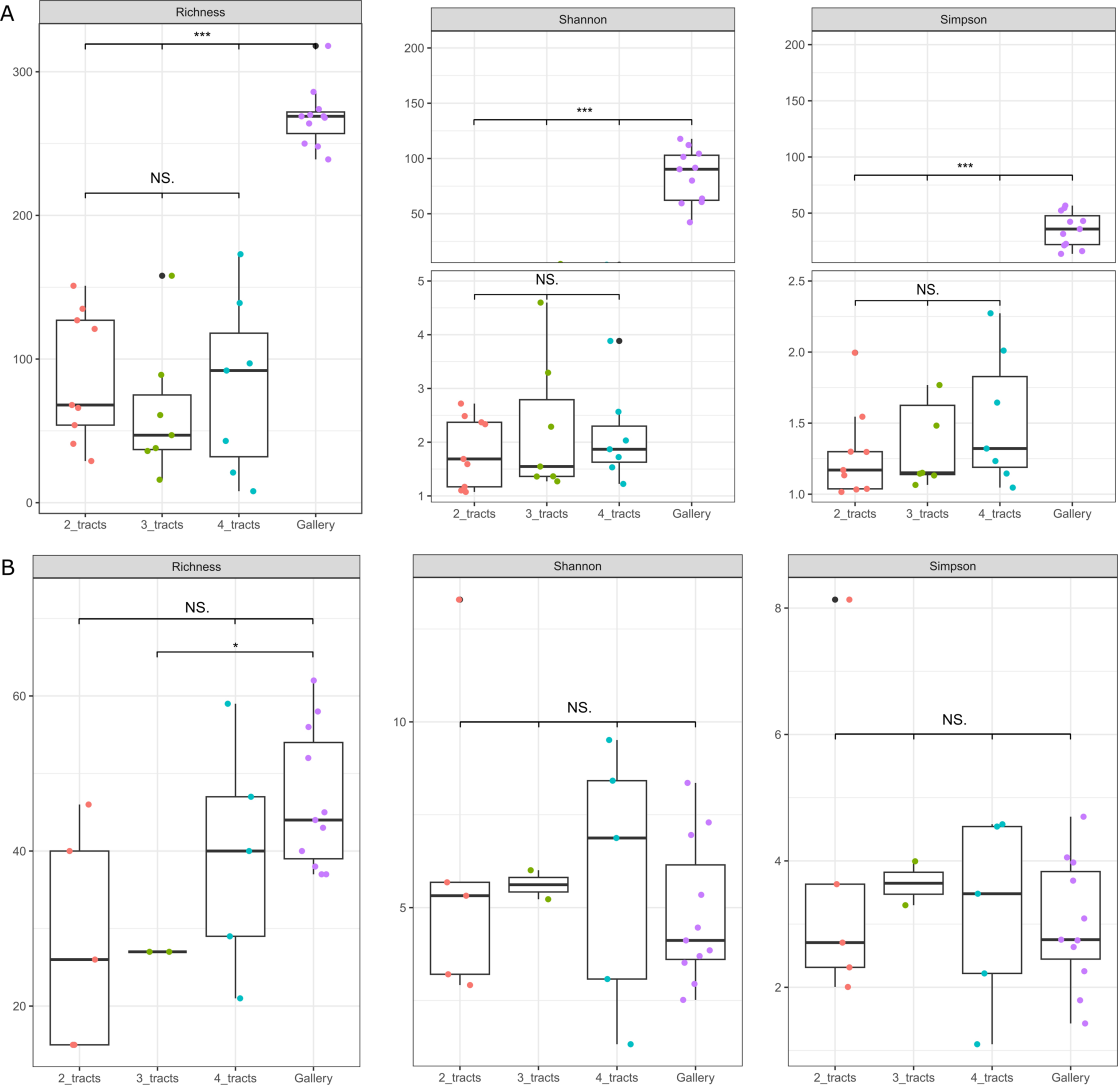
Box plots representing the alpha diversity distribution of the three Hill’s number (Richness, Shannon, and Simpson) for the galleries (purple) and the insect gut composite comprising 2 intestinal tracts (orange), 3 intestinal tracts (green) and 4 intestinal tracts (blue). **(A)** Alpha diversity of bacterial communities. **(B)** Alpha diversity of fungal communities. Significance levels between groups are indicated with a star (^*^p < 0.1, ^**^p < 0.01, and ^***^p < 0.001) or NS. if it is not significant.

The number of beetles found in cones mostly fluctuate between 3 and 7 individuals (Supplementary Table 2), enabling the integration of 2, 3 or 4 tracts of *C. coniperda* in composite samples prior to DNA extraction. The number of insects assembled in composite samples caused no significant incidence on the coverage of alpha diversity for bacterial and fungal communities (*p* > 0.3, *β* < 4%, Figure 3A). Galleries were also considered in the analysis as they are the immediate environment of the white pine cone beetle. The alpha diversity of bacterial communities in galleries was higher than observed in insects, regardless of the number of tracts considered (*p* < 0.0001). That dichotomy was less important for fungi. The alpha diversity of fungal communities in the gut was indistinguishable from the galleries (*p* > 0.2, *β* < 2%, Figure 3B). Trees from where the cones were collected did not have an influence on the bacterial and fungal alpha-diversity (*p* > 0.6). However, the experimental area explained variation of fungal diversity in galleries or in composite samples comprising 4 intestinal tracts (*p* < 0.05).

#### 3.2.2 Beta diversity

**Figure 4:**
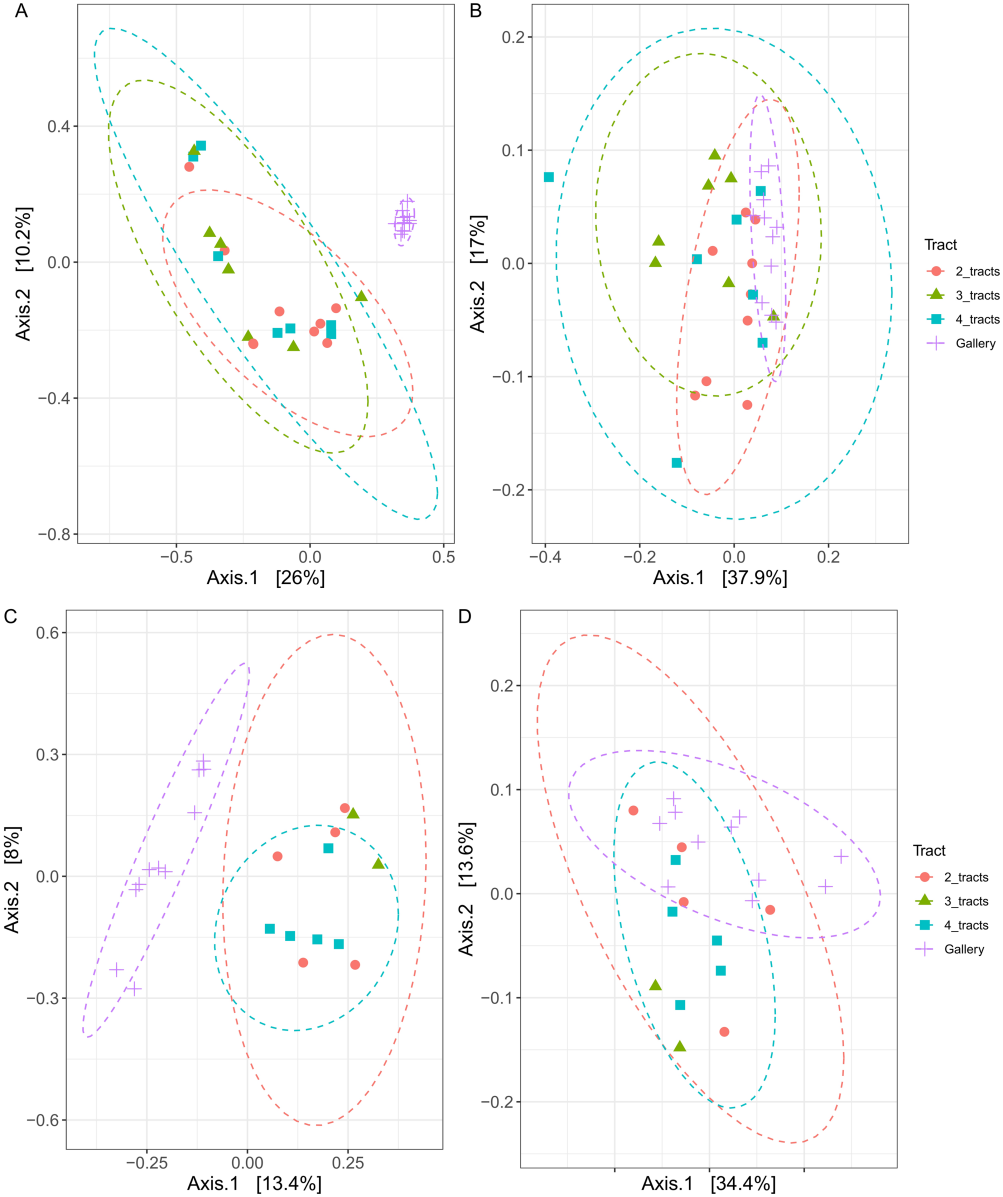
Principal coordinates analyses (PCoA) plot clustering the bacterial **(A,B)** and fungal **(C,D)** communities associated with *Conophthorus coniperda*’s gut and their galleries. Either Bray–Curtis **(A,C)** or weighted UniFrac **(B,D)** coefficients were used as a measure of dissimilarity or similarity between bacterial or fungal communities. Orange circle: 2 intestinal tracts; green triangle: 3 intestinal tracts; blue square: 4 intestinal tracts and purple cross: gallery.

**Figure 5:**
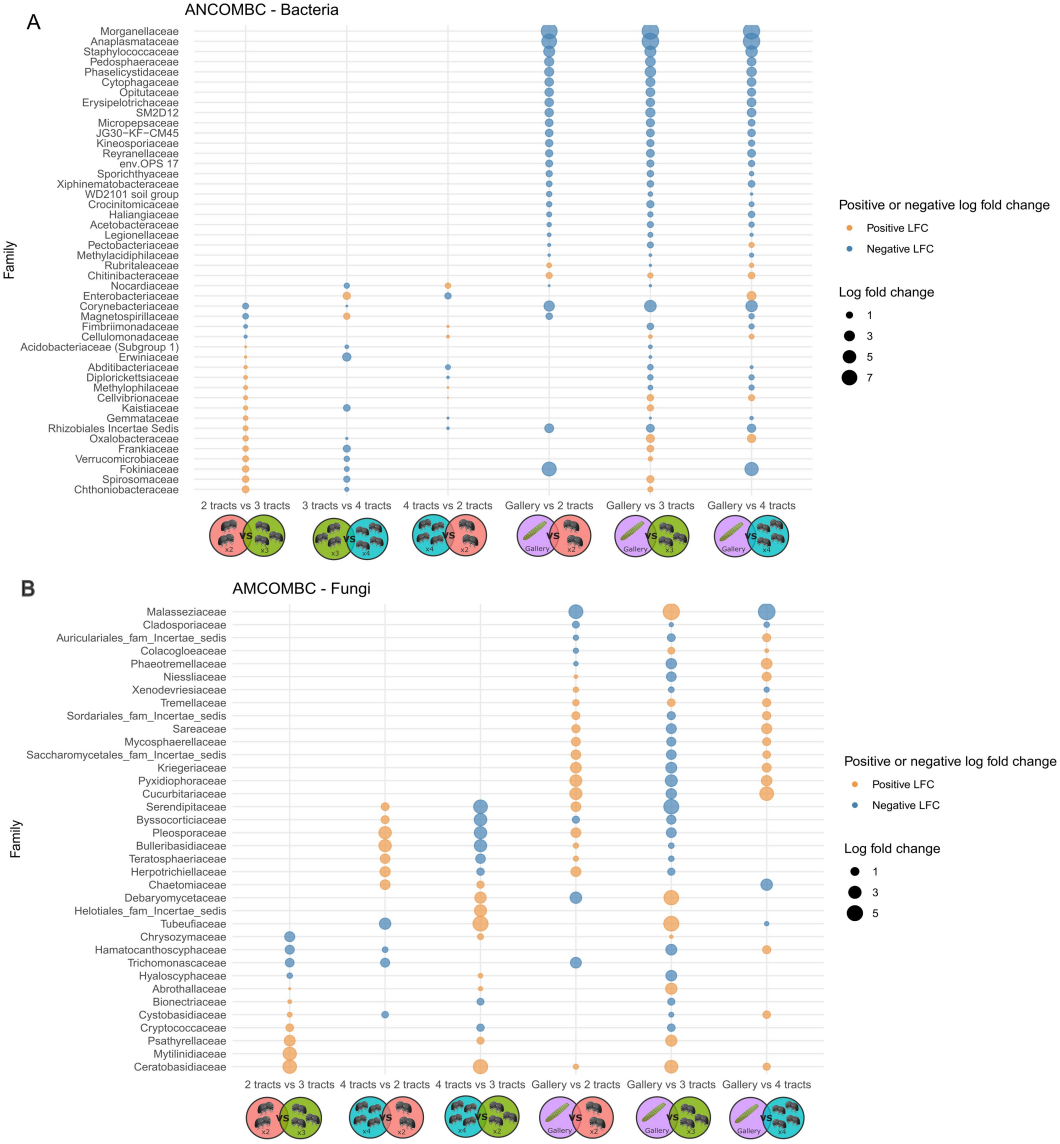
Graphical representation of analysis of compositions of microbiomes with bias correction (ANCOM-BC) clustering dual comparison of the bacterial **(A)** and fungal **(B)** communities of either 2 tracts (orange and 2 insects), 3 tracts (green and 3 insects), 4 tracts (blue and 4 insects) or galleries (purple and cone). The first group in each pair is used as the reference for each comparison. The positive and negative log fold changes always refer to the reference. An absence of bubble in the graph refer to no differences. The figure have been created using Biorender’s icons (https://BioRender.com/g02q920).

Variation of bacterial composition was not explained by the number of intestinal tracts included in composite samples, not withstanding the distance metric used (Figure 4, *p* > 0.6, *r*^2^ = 0.08). The relative abundance of 26 to 36 bacterial genera varied in composite samples comprising different number of tracts (Figure 5A). The composition of the tracts and their galleries are different (*p* < 0.007, *r*^2^ > 0.18). The distribution of 78 to 109 bacterial genera was distinct among tracts and galleries, depending on the number of tracts integrated in composite samples. Neither the block, nor the tree from where the samples were collected or the number of cones used influenced the composition of bacterial communities (*p* > 0.1, *r*^2^ > 0.02).

The composition of fungal communities was indistinguishable among the three classes of insect gut composites (*p* > 0.4, *r*^2^ = 0.2) neither the experimental area nor the trees had an influence on the fungal diversities, regardless of the distance’s metric used (*p* > 0.6, *r*^2^ = 0.1). Four tracts were necessary to observe a difference of fungal community with galleries (*p* < 0.04, *r*^2^ > 0.05). The relative abundance of 9 to 15 fungal genera varied in composite samples comprising different number of tracts (Figure 5). In comparison, the distribution of 29 to 36 bacterial genera was distinct among tracts and galleries, depending on the number of tracts integrated in composite samples. Overall, it does not seem to be any bacterial or fungal pattern arising from the number of intestinal tracts used.

### 3.3 How many more do we need?

**Figure 6:**
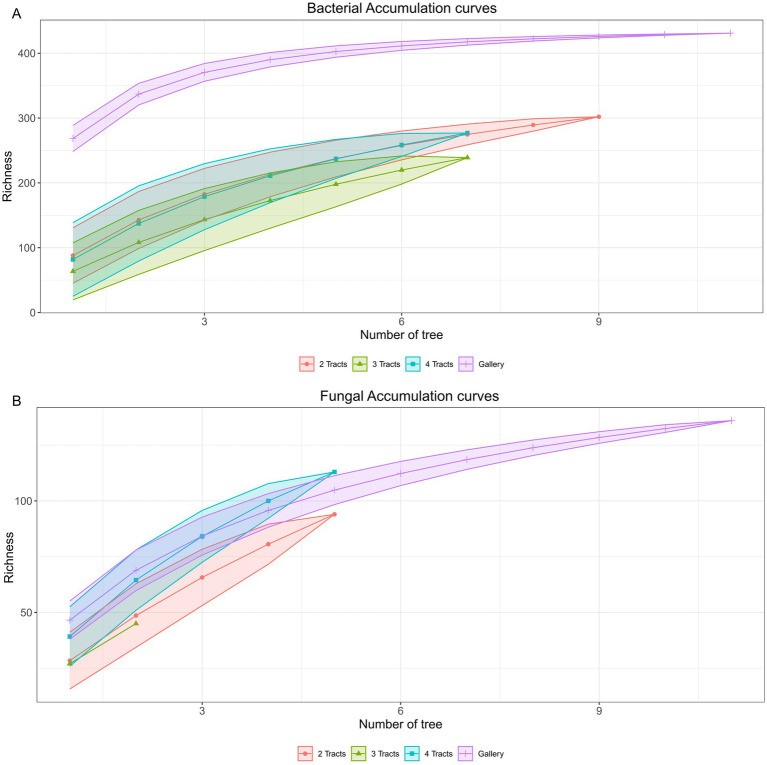
Accumulation curve of microbial communities obtain through the sampling of trees. **(A)** Accumulation curves of bacterial communities. **(B)** Accumulation curves of fungal communities. Orange circle: 2 intestinal tracts; green triangle: 3 intestinal tracts; blue square: 4 intestinal tracts and purple cross: gallery.

The gamma diversity of the microbiome of the insect in the experimental areas was examined at two levels: the number of trees and the number of guts integrated in composite samples. Total genomic DNA was insufficient for sequencing a few samples, leading to variable numbers of trees representing guts sample classes (Supplementary Table 2). Hence, the bacterial communities retrieved from samples comprising 2 guts were represented by 9 trees, whereas gut sample classes comprising 3 and 4 tracts were represented by 7 trees. Bacterial communities derived from 9 trees with 2 intestinal tracts, led to 76% coverage of the gamma diversity (Figure 6A), whereas 69% (±2.5) were achieved for the 7 trees. The bacterial diversity of the galleries was more homogeneous as 7 trees led to 93% recovery of gamma diversity, whereas the recovery increased to 95% with 9 trees. The fungal diversity was more heterogeneous than bacteria (Figure 6B). Integration of 7 trees led to recovery of 71% gamma diversity in the galleries whereas 77% was achieved with 9 trees. For the insects, the maximum number of trees sampled was 5 and allowed 63% (±7.8) recovery, which is similar to the recovery of bacterial gamma diversity achieved with the same number of tree (58% ± 2.1). Based on the model, 33 trees out of 1,046 are required to reach 95% of the bacterial and fungal diversity in the experimental area.

## 4 Discussion

The insect microbiota plays a crucial role in its adaptation to diverse environments. Studying how microorganisms contribute to host fitness and how environmental factors influence the structure of microbiota necessitates assessing the anticipated magnitude of both spatial and individual effects. The spatial effect has received attention and highlighted the contribution of dispersal as a significant contributor to insect microbiome structure (Paddock et al., 2022). Here, the number of insect guts integrated in composite samples prior to DNA extraction and the number of surveyed trees were expected to induce variability in recovered microbiome profiles.

The alpha and beta diversity were overall insensitive to the number of intestinal tracts integrated in composite samples. The distribution of a few ASV clustered at the genus level varied with the number of tracts integrated in composites. These differences were noticed using ANCOM-BC, considering the sampling fraction, i.e., the ratio between the expected absolute abundance of a sample and the absolute abundance of an ecosystem (Lin and Peddada, 2020). It is also worth noting that no clear pattern emerged as the number of intestinal tracts increases or decreases for these ASV clustered at the genus levels. Such a lack of trend either suggests that differences were caused by individual variability, feeding status or other abiotic and biotic determinants rather that the number of tracts used (Landry et al., 2022; Engel and Moran, 2013; Cole et al., 2021). A greater consistency between the response of genera distribution pattern and the number of tracts with composite samples comprising more than 4 insects can not be excluded at this stage. The effect size distinguishing the distribution of individual genotypes among replicated composite samples of insects will need to be considered in future studies examining the environmental drivers of insect microbiome.

The bacterial communities detected in galleries were statistically distinct from those retrieved from insects. This statistical distinction might be due to the specific requirement expected for the insect’s survival. For instance, one of the main family only found in insect, *Morganellaceae*, seems to be a vertically transmitted symbiont widely found in insect (Wierz et al., 2024). On the other hand, two of the main families present in the galleries, *Sphingomonadaceae* and *Pseudomonadaceae*, might provide protection and alimentation to the WPCB by degrading aromatic compounds or secreting antimicrobial substances (Ninkuu et al., 2021; Gershenzon and Dudareva, 2007; Micales et al., 1994; Teoh et al., 2021; Pessotti et al., 2021). It is worth noting that even if the bacterial communities statistically differ between the WPCB and the galleries, the main bacterial families of found in galleries are also present in insect’s tract, in lower abundance (Figure 2; Supplementary Table 2). In insect, essentials microorganisms are generally vertically transmitted while the facultative one are acquired horizontally through feeding or contact with the environment (Coolen et al., 2022). They could be obtained through coprophagy as insect frass can act as a transmission route for microorganism (Jahnes et al., 2019; Mitchell and Hanks, 2009). In the case of the WPCB, it would be necessary to assess the microbial charge of non-attacked cone to evaluate the origin of those microorganisms. It would help us understand if the latter generation inoculated the cone or if it was acquired from the cone. In addition, to fully capture the function of the bacterial communities and to ensure the taxonomic tendency, it would be of interest to do some metagenomic shotgun sequencing.

Fungal communities displayed higher richness in galleries, but their beta diversity was comparable with the diversity of galleries. Some wood-boring insects, such as ambrosia beetles, are known to feed on fungi with which they generally have a symbiotic relationship (Hulcr and Stelinski, 2017). They are known for gardening by harboring microorganisms that will grow in their environment and bring them all the nutriment needed and otherwise absent (Diehl et al., 2022). As a results, fungi are often at the core of their alimentation (Stefanini, 2018). Similar fungal communities in the intestinal tract of the WPCB and galleries suggest a consistent association of certain fungal species with *C. coniperda* inside cones. The *Nectriaceae* family, which is the main common family found, include the fungal genus *Fusarium*, known as an ambrosial mutualist (Kasson et al., 2013; Lynch et al., 2024). However, potential gardening behavior is rare or even absent for other wood-boring insects. In particular, WPCB is a bark beetle, i.e., beetles commonly feeding on the phloem or other tree parts (e.g., cone in this case) and less dependent on fungal associates. In this sense, either the fungi similarity found between the WPCB’s intestinal tract, and the cones is due do the sole presence of the fungi in the galleries, or this species could present specific symbiotic relationships, like *Dendroctunus* spp. for example (Harrington et al., 2005; Bateman et al., 2016). To decipher the implications of these fungi in WPCB bio-ecology, further analysis should be performed.

The analyses of the gamma diversity suggested that the sampling of 33 trees out of 1,046 in 1.1 ha is required to cover 95% of the bacterial and fungal diversity in the two experimental areas of the white pine seeds orchard. This estimate was extrapolated from a maximal number of five fungal samples and 9 bacterial samples, which is prone to potential overestimation of the number of sample needed up to 20% (Chao et al., 2009). Even though the coverage percentage is the same for both the bacterial and the fungal diversity when 5 trees are considered (60% ± 4.7), the number of samples needed to reach 95% of the fungal diversity is 1.5 time higher than needed for bacterial diversity. Following the hypothesis of a potential overestimation when fewer sample are used and basing the calculations on bacterial results, we might only need 21 samples to cover 95% of microbial diversity and 28 samples to cover 98%. However, it is worth noting that fungal communities seem to be harder to cover and need a more extensive sampling effort to reach a similar coverage (Supplementary Table 2).

## 5 Conclusion

This is the first study of *C. coniperda* microbiome and the (Harrington et al., 2005) one associated with the direct environment of the insect. A contrasting bacterial microbiome was observed between insect and galleries, but a surprisingly similar one for the fungal microbiome. These observations raised more questions than answer, offering multiple research opportunities to study the relation between the WPCB’s microbiome and its environment. On the other hand, the variation of microbiome composition at the orchard, tree and insect level will guide future investigations seeking to relate environmental variables to *C. coniperda* microbiome structure. The number of guts included per composite was comprised within a narrow range, excluding individual gut due to PCR amplification constraints. Nevertheless, minimal sample sizes of 4 intestinal tracts per experimental unit and 28 to 33 trees in an area of 1.1 ha are recommended to relate insect microbiome to climate and landscape features.

## Data Availability

The datasets presented in this study can be found in online repositories. The names of the repository/repositories and accession number(s) can be found at: https://www.ncbi.nlm.nih.gov/bioproject/PRJNA1146082, PRJNA1146082.
