# Supplementary material for: How many do we need? Meeting the challenges of studying the microbiome of a cryptic insect in an orchard
Source: Front Microbiol. 2025 Jan 6;15:1490681. doi: 10.3389/fmicb.2024.1490681 (PMC11743375; doi:10.3389/fmicb.2024.1490681)
Supplement: Supplementary file 1 [file Image_1.pdf]

Supplementary Material

1.1 Supplementary Figures

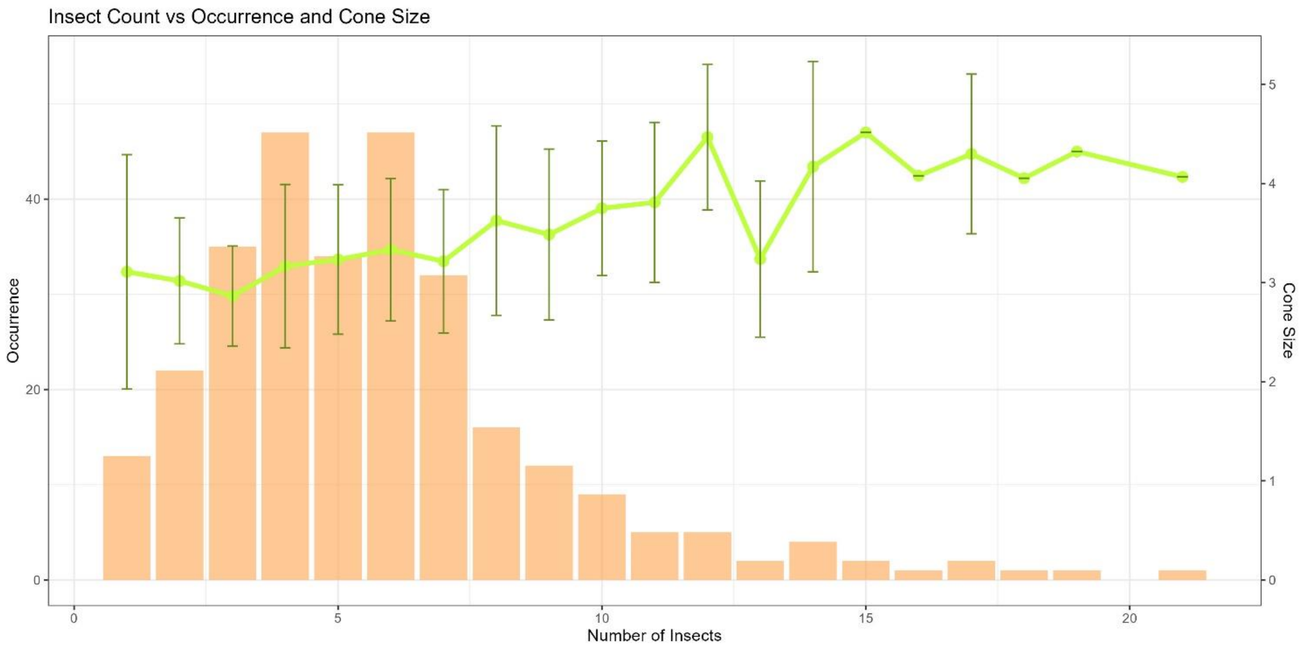

Supplementary Figure 1. Occurrence of the number of insects throughout 292 cones (Barplot) and it's relation with the size of the cone (Lineplot).
